# Defensin-Like ZmES4 Mediates Pollen Tube Burst in Maize via Opening of the Potassium Channel KZM1

**DOI:** 10.1371/journal.pbio.1000388

**Published:** 2010-06-01

**Authors:** Suseno Amien, Irina Kliwer, Mihaela L. Márton, Thomas Debener, Dietmar Geiger, Dirk Becker, Thomas Dresselhaus

**Affiliations:** 1 Plant Breeding Laboratory, University of Padjadjaran, Bandung, Indonesia; 2 Cell Biology and Plant Biochemistry, University of Regensburg, Regensburg, Germany; 3 Institute for Plant Genetics, Molecular Plant Breeding, Leibniz University of Hannover, Hannover, Germany; 4 Plant Molecular Physiology and Biophysics, University of Würzburg, Würzburg, Germany; Temasek Life Sciences Laboratory, Singapore

## Abstract

In contrast to animals and lower plant species, sperm cells of flowering plants are non-motile and are transported to the female gametes via the pollen tube, i.e. the male gametophyte. Upon arrival at the female gametophyte two sperm cells are discharged into the receptive synergid cell to execute double fertilization. The first players involved in inter-gametophyte signaling to attract pollen tubes and to arrest their growth have been recently identified. In contrast the physiological mechanisms leading to pollen tube burst and thus sperm discharge remained elusive. Here, we describe the role of polymorphic defensin-like cysteine-rich proteins ZmES1-4 (*Zea mays* embryo sac) from maize, leading to pollen tube growth arrest, burst, and explosive sperm release. *ZmES1-4* genes are exclusively expressed in the cells of the female gametophyte. ZmES4-GFP fusion proteins accumulate in vesicles at the secretory zone of mature synergid cells and are released during the fertilization process. Using RNAi knock-down and synthetic ZmES4 proteins, we found that ZmES4 induces pollen tube burst in a species-preferential manner. Pollen tube plasma membrane depolarization, which occurs immediately after ZmES4 application, as well as channel blocker experiments point to a role of K^+^-influx in the pollen tube rupture mechanism. Finally, we discovered the intrinsic rectifying K^+^ channel KZM1 as a direct target of ZmES4. Following ZmES4 application, KZM1 opens at physiological membrane potentials and closes after wash-out. In conclusion, we suggest that vesicles containing ZmES4 are released from the synergid cells upon male-female gametophyte signaling. Subsequent interaction between ZmES4 and KZM1 results in channel opening and K*^+^* influx. We further suggest that K*^+^* influx leads to water uptake and culminates in osmotic tube burst. The species-preferential activity of polymorphic ZmES4 indicates that the mechanism described represents a pre-zygotic hybridization barrier and may be a component of reproductive isolation in plants.

## Abstract 2

Species-preferential osmotic pollen tube burst and sperm discharge in maize involve induced opening of the pollen tube-expressed potassium channel KZM1 by the egg apparatus-derived defensin-like protein ZmES4.

## Author Summary

Sperm cells of animals and lower plants are mobile and can swim to the oocyte or egg cell. In contrast, flowering plants generate immobile sperm encased in a pollen coat to protect them from drying out and are transported via the pollen tube cell towards the egg apparatus to achieve double fertilization. Upon arrival the pollen tube tip bursts to deliver two sperm cells, one fusing with the egg cell to generate the embryo and the other fusing with the central cell to generate the endosperm. Here, we report the mechanisms leading to pollen tube burst and sperm discharge in maize. We found that before fertilization the defensin-like protein ZmES1-4 is stored in the secretory zone of the egg apparatus cells and that pollen tubes cannot discharge sperm in *ZmES1-4* knock-down plants. Application of chemically synthesized ZmES4 leads to pollen tube burst within seconds in maize, but not in other plant species, suggesting this mechanism may be species specific. Finally, we identified the pollen tube-expressed potassium channel KZM1 as a target of ZmES4, which opens after ZmES4 treatment and probably leads to K^+^ influx and sperm release after osmotic burst.

## Introduction

**Figure 1:**
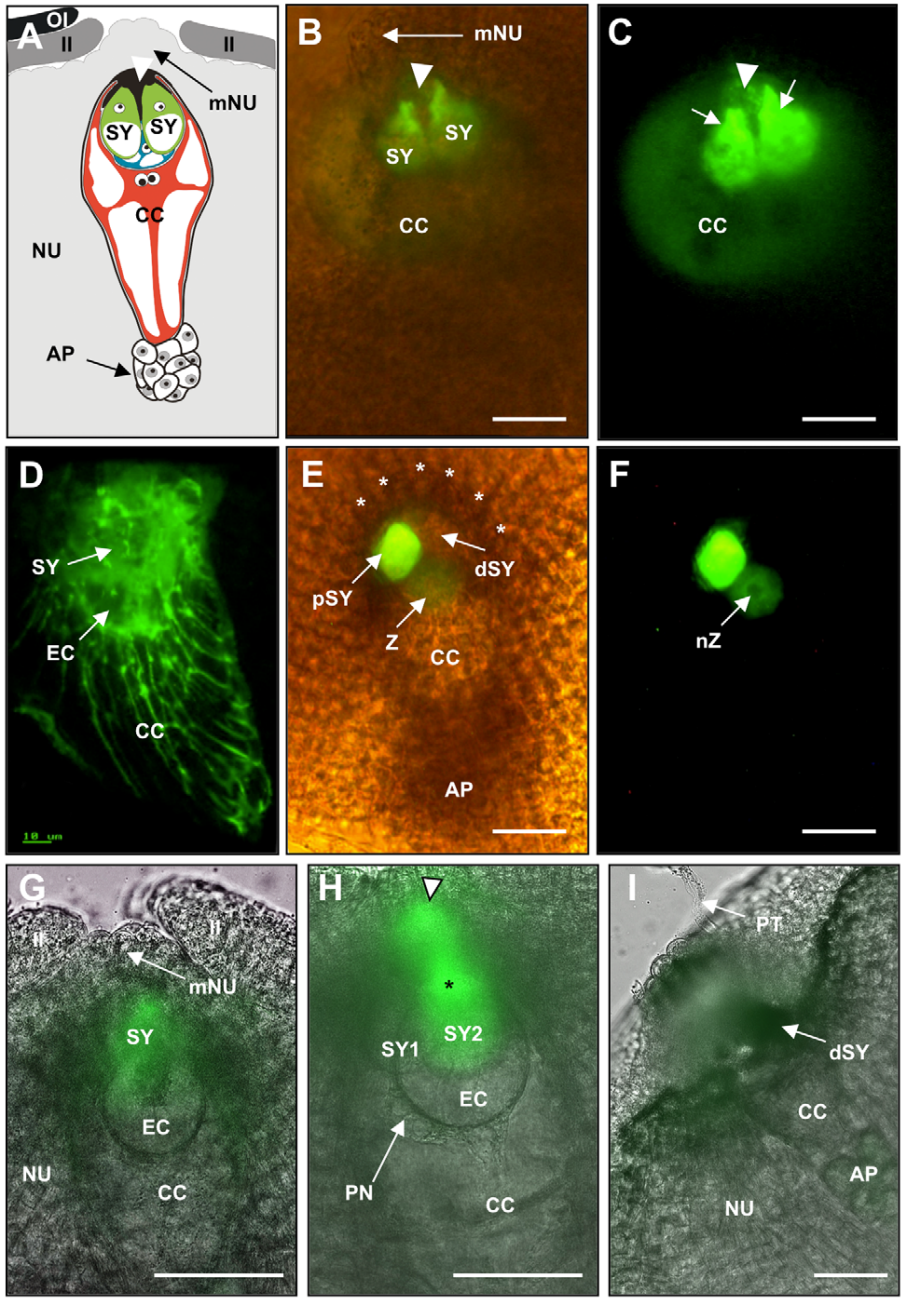
ZmES4 is predominately localized to the secretory zone of the mature maize egg apparatus before fertilization. (A) Diagram of the female gametophyte (embryo sac) of maize embedded in the maternal tissues of the ovule. Outer integument is black, inner integument dark grey, and nucellus light grey. The egg cell (blue) is hidden behind the two synergids and the filiform apparatus is indicated with a white arrowhead. (B) Merged bright-field and UV micrograph showing a slanting oversight of the micropylar region. The inner integument has been removed. ZmES4-GFP fusion protein under control of the endogenous promoter is localized to the synergid cells with strongest signals around the filiform apparatus (arrowhead). The arrow points towards the five to six micropylar nucellus cell stacks enclosing the egg apparatus. (C) Enhanced UV image of (B) to show strongest GFP signals in the secretory zone of the two synergid cells (arrows). Vesicles are visible in the synergid extensions within the filiform apparatus (arrow head). Weaker signals are visible in the central cell. (D) CLSM image stack of 30 1 µm sections of a young mature embryo sac. The ZmES4-GFP fusion protein is visible in cytoplasmic strands probably resembling the ER and vesicles of the egg apparatus (egg cell and synergids) and central cell but not in the antipodals. Highest signals are visible in the egg apparatus. (E) Merged bright-field and UV micrograph showing a section along the embryo sac 15 h after in vitro pollination with a view from the inside of the ovule. The surface of micropylar nucellus cell stacks is indicated by asterisks. (F) UV image of (E) to show that GFP signals are no longer visible in the cell wall of the egg apparatus, in the receptive and degenerated synergid cell, as well as in the central cell. Strongest signal is detectable in the persisting synergid cell and weaker signals around the nucleus of the zygote. 24 h after in vitro pollination, signals were no longer detectable (unpublished data). (G–I) Longitudinal sections of a marker line displaying GFP only in one of the two synergid cells. View from the inside of the ovule towards the female gametophyte. (G) A young embryo sac shortly after cellularization displays GFP signals exclusively in the synergid cell. (H) A mature embryo sac of the same line shows strong GFP signals around the nucleus (asterisk) and in the secretory zone (arrowhead) of one synergid cell. (I) The GFP expressing synergid cell is the exclusive recipient of pollen tubes, indicated by the loss of GFP signal. AP, antipodals; CC, central cell; EC, egg cell; II, inner integument; mNU, micropylar nucellus; NU, nucellus; OI, outer integument; SY, synergid; dSY, degenerated synergid; pSY, persisting synergid; PN, polar nuclei of the central cell; PT, pollen tube; Z, zygote; nZ, nucleus of zygote. Scale bars are 50 µm.

Flowering plants (angiosperms) emerged some 180–140 MYA [1] and have since inhabitated most ecological environments, which are often far away from humid conditions. Hence, new reproductive mechanisms were acquired to keep cells from drying out and to realize species-specific interactions between male and female reproductive structures, partly over long distances. Adaptive selection led to the reduction of the haploid male gametophyte to a three-cellular pollen grain and pollen tube, respectively, which is able to be transported over long distances and to grow deeply inside female reproductive tissues. As a further consequence the sperm cells lost their motility. The reduced female gametophyte (embryo sac), which is haploid in most plant species, is deeply embedded and protected in the maternal tissues of the ovule and ovary and harbors the female gametes (egg and central cell) as well as some accessory cells (synergid and antipodal cells; Figure 1A). Of these, the synergid cells are involved in pollen tube signaling, sperm delivery, and transport [2]. Extensive cell-cell communication events are likely to take place between both gametophytes, among male and female gametophyte cells, respectively, as well as the surrounding sporophytic tissue [3]. The central events preceding fertilization involve signaling towards the pollen tube to arrest growth and to induce discharge of the two sperm cells, a process first described by the famous German-Polish botanist Eduard Strasburger in 1884 [4]. It took more than 120 years to identify the first molecular players involved in these processes.

Cross-talk between both gametophytes to arrest pollen tube growth has recently been shown to depend on the FER receptor-like kinase (RLK) [5] and the GPI-anchored protein LRE [6] localized at the synergid plasma membrane. Nitric oxide and reactive oxygen species seem to play additional roles in this process [7],[8], and the activity of the Ca^2+^ pump ACA9 [9] localized at the pollen tube plasma membrane was shown to be required for pollen tube rupture. We were investigating whether small secreted cysteine-rich proteins (CRPs), specifically expressed in the embryo sac cells, play a role in these processes. Plant genomes encode large classes of CRPs accounting for more than 800 genes in Arabidopsis and approximately 600 genes in rice. Among them, defensins/defensin-like proteins (DEFs/DEFLs), lipid transfer proteins (LTPs), rapid alkanization factor (RALF) proteins, and thionins resemble the largest classes [10]. The majority of these genes are expressed in reproductive tissues and a number of genes have been shown to be specifically expressed in the cells of the embryo sac [11],[12],[13],[14],[15],[16]. Here, we report the functional analysis of a small gene-family of four members encoding DEFL proteins in the maize inbred line A188. All four genes are specifically expressed in the embryo sac and have been shown previously to be down-regulated immediately upon fertilization [12].

## Results

### ZmES4 Accumulates in the Secretory Zone of the Synergid Cells

ZmES1-4 protein localization was studied in the maize embryo sac cells before and after fertilization in plants expressing GFP fused to the C-terminus of ZmES4 under control of its endogenous promoter. All transgenic lines showed fusion protein localization exclusively in the embryo sac cells. Some variation was observed among the cell types expressing ZmES4-GFP: most ovules expressed it in both synergid cells, others in about 80% ovules only in one synergid cell, while others showed expression additionally in egg and central cells. Signals in the antipodal cells were never observed. As shown in Figure 1A–1C and Figure S1N and S1O, the fusion protein was most prominently located in the secretory zone of the two synergids surrounding the filiform apparatus before fertilization. Unlike the ZmEA1-GFP protein, which is secreted from the egg apparatus and plays a role in short range pollen tube guidance [17], GFP fluorescence was not detected in the cell walls of micropylar nucellus cells. Weaker signals were visible in the central cell of some but not all transgenic lines. In a stack of confocal laser scanning microscopy images (Figure 1D) of a very young cellularized embryo sac, displayed fusion protein signals were also found in the endoplasmic reticulum (ER) of the central cell. 15 hours after pollination (hap), around 7 to 8 h after fertilization, ZmES4-GFP was no longer detectable in the degenerated receptive synergid cell and polar localization in the persisting synergid cell was lost (Figure 1E and 1F). Faint fluorescence, accumulating in the ER around the nucleus, was visible in the fertilized egg cell of some transgenic lines. The fusion protein was no longer detectable in all lines 24 hap, indicating that it was actively degraded after fertilization. Time course measurements using a line expressing the fusion protein in about 80% ovules only in one of the two synergid cells showed that signals first appeared after the whole embryo sac is fully differentiated (Figure 1G). During further maturation and embryo sac enlargement, most fluorescence was visible in the ER around the nucleus and the secretary zone of the synergid cell (Figure 1H). In this line, the single ZmES4-GFP expressing synergid cell was the exclusive target of the pollen tube (Figure 1I). Transient transformation of onion epidermis cells was used to investigate the secretion of ZmES proteins. As shown in Figure S1, ZmES4-GFP fusion protein entered the secretory pathway and seemed to be present in golgi stacks, constitutive secretory vesicles, as well as in the cell wall. We suggest, however, that in contrast to onion epidermis cells, in non-degenerate synergid cells, ZmES4-GFP appears to be retained in regulated secretory vesicles, as signals were not observed in the cell wall before fertilization.

### ZmES4 Has Low DEF Activity

In order to study the DEF activity of ZmES proteins, *ZmES4* was expressed under the control of the ubiquitously expressed *35S* promoter in *Arabidopsis*. As shown in Figure S2A and S2B, antibacterial activity, which has also been reported for most plant DEFs studied so far [18], was not observed. Although antifungal activity was also not observed at the seedling stage (Figure S2C), infected ZmES4 over-expressing seedlings recovered faster than control seedlings and fungal hyphae were no longer macroscopically visible a few weeks after infection. In contrast, control plants still contained fungal hyphae, displayed chlorosis, and delayed flowering (Figure S2D). These experiments indicated that ZmES proteins still display low DEF activity and are apparently able to bind to fungal targets with a low affinity. Their major physiological function, however, is likely to be different.

### ZmES Function Is Required for Pollen Tube Growth Arrest and Burst

**Figure 2:**
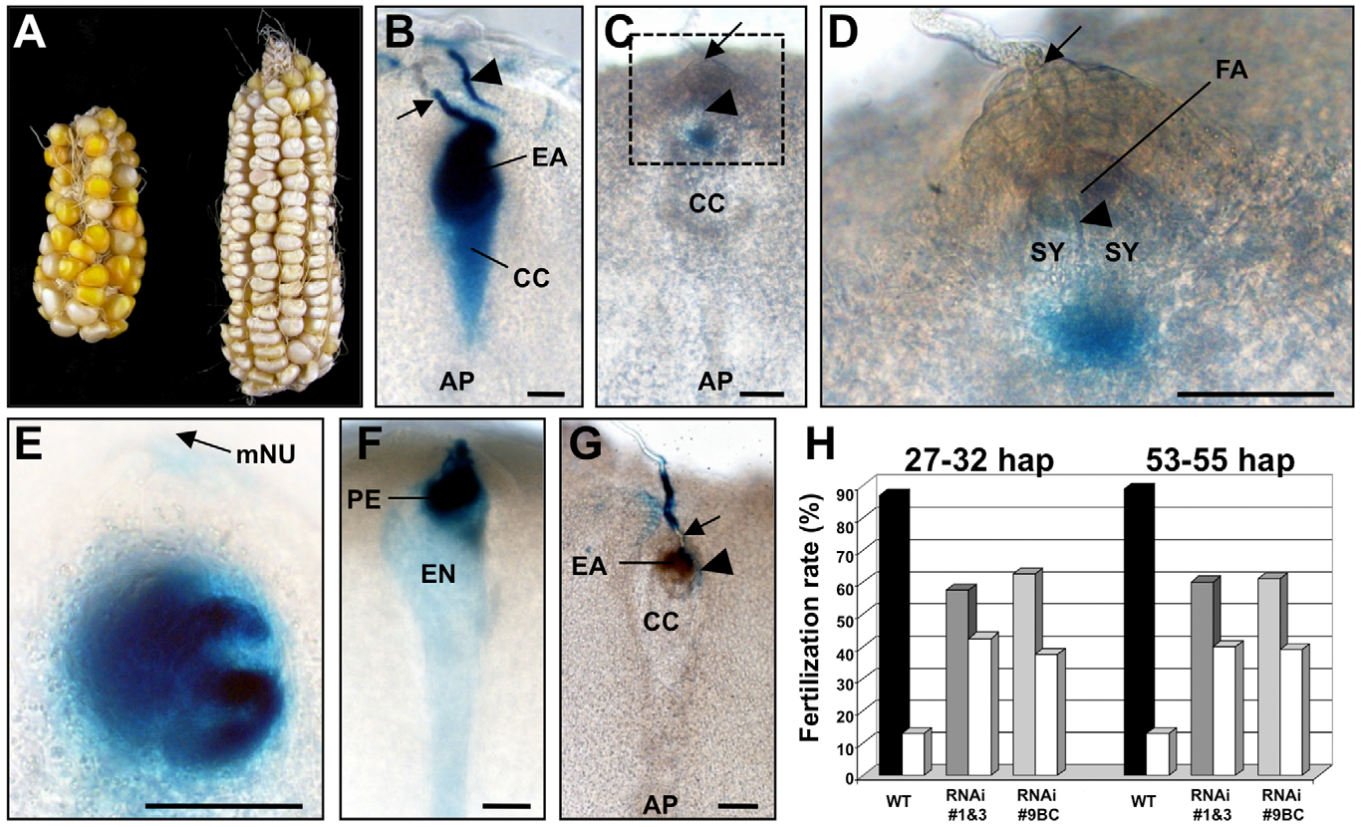
Pollen tube arrest and burst is impaired in transgenic lines with reduced *ZmES* activity. (A) In order to study the function of *ZmES* genes, *ZmES4-*RNAi mutants were generated to down-regulate the whole *ZmES* gene-family simultaneously by RNA silencing. Only about half of self-pollinated ovules of *ZmES4-*RNAi lines developed into kernels (left). Pollination of wt (A188) ovules using pollen from *ZmES4-*RNAi plants led to full seed set (right) and about 43% transmission of a single copy RNAi construct in progeny plants (unpublished data). (B–G) *ACT*p*:GUS* maize pollen was used to monitor pollen tube growth and fertilization rates inside maize ovaries between 27–32 hap (B–E) and 72 hap (F and G), respectively. (B) One pollen tube (arrow) has penetrated the egg apparatus of a wt ovule. Blue staining of both egg apparatus and central cell indicates successful double fertilization. A second pollen tube (arrowhead) is no longer directed towards the fertilized egg apparatus. (C) An *ACT*p*:GUS* maize pollen tube has penetrated the nucellar tissue (arrow) and female gametophyte (arrow head) of an *ZmES4-*RNAi ovule, but GUS signals are neither detectable in the synergid nor in the central cell. (D) A close-up of (C) shows that the pollen tube grew into the cell wall material of the filiform apparatus between the two synergids (arrowhead). GUS activity is visible inside the female gametophyte in the region between egg and central cell indicating pollen tube growth arrest. (E) Another example of an *ZmES4-*RNAi ovule containing a pollen tube inside the egg apparatus region lacking both growth arrest and burst. (F) A wt ovule shows paternal GUS-activity in both pro-embryo and developing endosperm. The endosperm has already enlarged and contains a large number of nuclei (not visible). (G) The nucellus tissue (arrow) of an *ZmES4-*RNAi ovule was penetrated by a pollen tube, which grew around the egg apparatus (arrowhead) without penetration. The synergid cells have already started to degenerate at this stage. (H) Fertilization rates of both egg and central cell are significantly impaired in *ZmES4-*RNAi ovules. On average 88% wt ovules are fertilized (black columns), compared to 60% fertilized *ZmES4-*RNAi ovules (grey columns). A difference between self-pollinated *ZmES4-*RNAi ovules as well as ovules from back-crossed (BC) plants was not detectable neither 1 nor 2 d after fertilization (27–32 and 53–55 hap, respectively). The percentage of unfertilized ovules for both wt and ES4-RNAi lines are represented by white columns. A detailed list of sectioned and analyzed ovaries is attached as Table S1. AP, antipodals; CC, central cell; EA, egg apparatus; EN, endosperm; FA, filiform apparatus; hap, hours after in vitro pollination; mNU, micropylar nucellus; PE, pro-embryo; SY, synergid. Scale bars are 50 µm.

To study the role of ZmES proteins during fertilization, the whole gene-family was down-regulated using RNAi-silencing. *ZmES4*-RNAi plants, which showed weak or lack of the RNAi-transcript, did not show any obvious phenotype. Plants that contained high RNAi-transcript levels displayed female sterility after self-pollination (Figure 2A) but full seed-set after back-crossing to wt plants. Progeny of a single copy RNAi-line back-crossed to wild type plants displayed 43% transmission of the transgene via the pollen (*n* = 28). Self-pollination showed 50% transmission (*n* = 30), indicating almost full transmission of the silencing construct via the pollen but low transmission via the female gametophyte.

After pollination with an *ACT*p:*GUS* marker line [17], maternal tissues of the ovary and ovule were sectioned to visualize the pollen tube during the fertilization process. As shown in Figure 2B, a wt pollen tube penetrated the embryo sac, released its content, and blue staining of both egg and central cell indicated successful fertilization due to the activity of sperm derived paternal *GUS* genes inside female gametes. After fertilization, guidance signals apparently no longer existed, as additional pollen tubes failed to penetrate the micropylar nucellus region of the ovule. In contrast, *ZmES4*-RNAi plants displayed penetration of the micropylar nucellus, but GUS signals were neither detectable inside the gametes nor inside the synergid cell (Figure 2C–2E). Moreover, both synergid cells were still intact and gamete delivery had not occurred. Pollen tube over-growth was partially observed inside the egg apparatus region (Figure 2E) or growth around the egg apparatus (Figure 2G). Here, the very mature egg apparatus cells started to disintegrate. Fertilized wt ovules of the same cob show enlargement of the embryo sac and initiation of embryo and endosperm development (Figure 2F). A summary of fertilization rates is shown in Figure 2H (see also Table S1): two *ZmES4*-RNAi-lines and a single copy back-crossed line were analyzed for occurrence of fertilization around 1 and 2d after fertilization (27–32 hap and 53–55 hap), respectively. GUS activity in both, egg and central cell, was measured as successful fertilization. In contrast to wt embryo sacs that displayed a fertilization rate of 88%, *ZmES4*-RNAi lines showed a reduction to 60%. This value is higher than the expected 44% of a genetic null-mutant indicating that either the silencing effect is incomplete and/or additional factors are involved in the release of pollen tube contents.

### ZmES4 Induces Pollen Tube Plasma Membrane Depolarization and Burst in a Species-Preferential Manner

**Figure 3:**
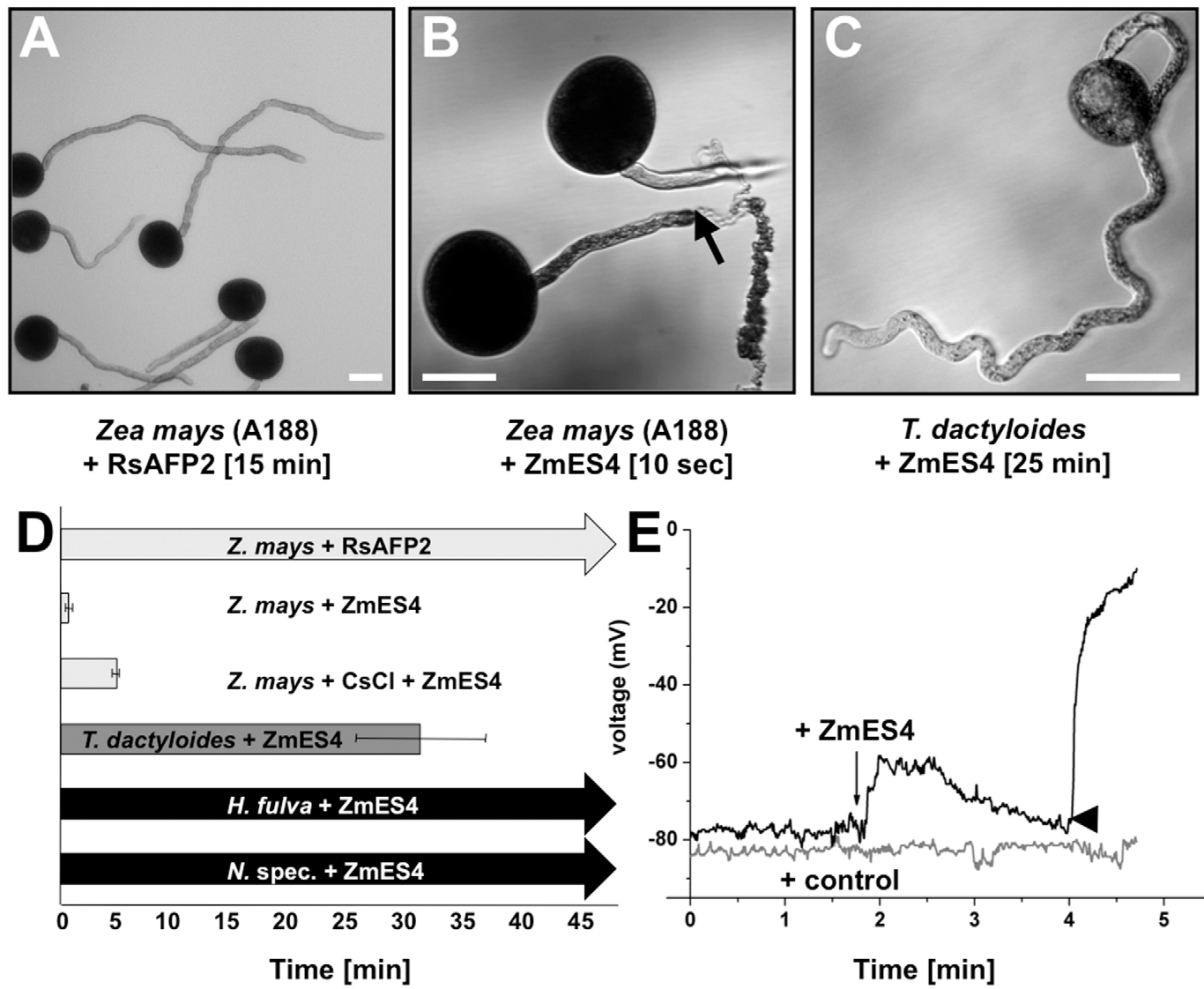
Mature defensin-like ZmES4 induces pollen tube tip burst after depolarization of the pollen tube membrane. In order to investigate the role of ZmES4 activity on living pollen tubes, an in vitro pollen tube growth bioassay using various plant species was established. (A) A pollen grain of maize (inbred A188) was germinated for 15 min in liquid medium before addition of 30 µM RsAFP2 in PGM. Growth behavior was further observed for up to 120 min (here after 15 min incubation) displaying normal tube growth. (B) Maize pollen grains were germinated for 15 min before addition of PGM containing 30 µM ZmES4. Pollen tubes started to burst within seconds after ZmES4 application and tube content was released explosively at the tip (arrow). (C) Pollen grains of *Tripsacum dactyloides* were germinated for 15 min before PGM containing 30 µM ZmES4 was added. 15 min after ZmES4 application most pollen tubes were still intact and grew further until rupture occurred on average around 32 min after incubation. (D) Diagram showing average time points when 80% of germinated pollen tubes of maize (*Zea mays*) and *T. dactyloides* were ruptured after ZmES4 protein application. Growth of pollen tubes of lily (*H. fulva*) and various tobacco species (*N.* spec.) observed for more than 60 min was not affected by ZmES4 application. CsCl_2_ application significantly delayed ZmES4 activity. Standard deviations are indicated. (E) Micro-electrode impalement of *Zea mays* pollen tubes for membrane potential recordings. Pollen grains of maize were germinated on solid medium and impaled right below the pollen tube tip. Control application of CRPs such as trypsin inhibitor from soybean or RsAFP2 in PGM did not affect the pollen tube membrane potential, while the application of ZmES4 in PGM evoked a transient depolarization of the membrane potential followed by pollen tube tip burst (arrowhead). A representative recording is shown displaying tube burst 2 min after ZmES4 application. Bars: 50 µm.

Due to limitations in visualizing the double fertilization process *in planta*, we chemically synthesized mature ZmES4 protein and investigated its effect on the growth behavior of in vitro in liquid-germinated and -grown pollen tubes of maize and other plant species. Buffer containing 30 µM ZmES4 induced rapid pollen tube burst at the very tube tip within seconds after application to germinated pollen tubes (Figure 3B). Within less than a minute almost 100% pollen tubes ruptured and tube content was released explosively. Even at 1,000-fold lower concentrations, one-third of pollen tubes bursted within less than 2 min (Figure S3). This reaction was species-preferential as pollen tube rupture in the maize relative *Tripsacum dactyloides* was essentially delayed (around 1,600–2,300 s versus 35–50 s in maize, independent from the maize genotype used; Figure 3C and 3D) or did not occur at all in other plant species tested, such as tobacco or lily (Figure 3D). In contrast, other extracellular CRPs, trypsin inhibitor from soybean [19], or LURE2 of *Torenia fournieri* [20] or AFP2 from *Raphanus sativus* [21] (Figure 3A) did not affect pollen tube growth behavior when applied at the same concentration and the same conditions. This indicates that maize and their relatives possess species-preferential ZmES targets.

Rapid pollen tube rupture pointed to an osmotic process and, consequently, altered solute transport at the pollen tube plasma membrane. In order to obtain a mechanistic clue about ZmES activity, the membrane potentials of growing pollen tubes were recorded, using the micro-electrode impalement technique. Membrane potentials in the range of −70 to −100 mV (*n* >10) were recorded in fast growing pollen tubes (average −80 mV). In contrast to control substances, ZmES4 application led to a transient membrane depolarization followed by repolarization efforts, but the burst generally occurred within 1 to 2 min before repolarization was achieved (Figure 3E). These measurements indicate that ZmES4 application either leads to Cl^−^ efflux or H^+^ and/or K^+^ influx and that ZmES4 target(s) likely represent ion-channel(s).

### ZmES4 Opens the Inward Rectifying Potassium Channel KZM1

**Figure 4:**
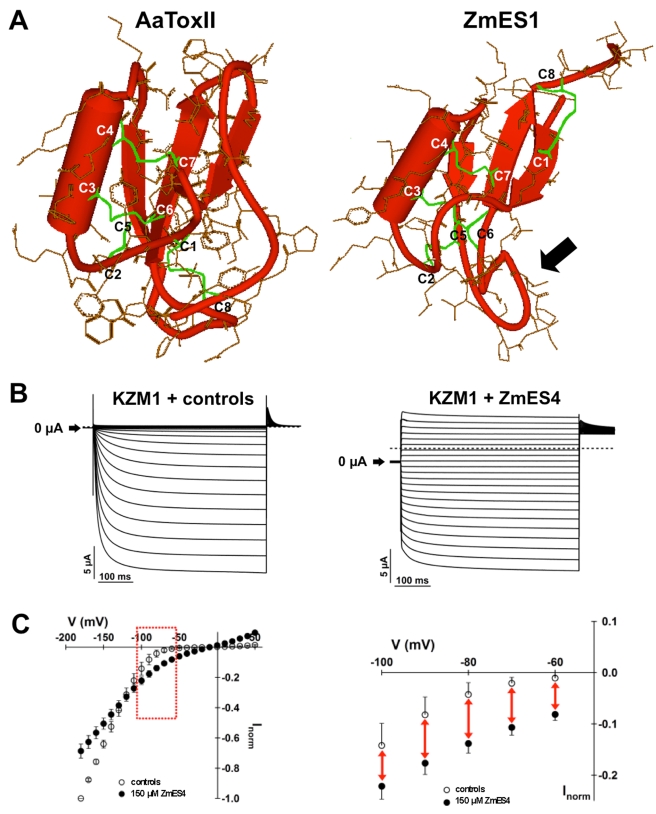
ZmES4 opens the pollen tube expressed potassium channel KZM1. (A) X-ray structure of Sahara scorpion (*Androctonus australis*) neurotoxin II (AaToxII: PDB code 1PTX; left) and predicted 3D-structure of ZmES1 of maize (right). The ZmES1 3D-structure was generated using the program MODELLER based on various defensin NMR structures. The structure is almost identical to that of ZmES4 (Figure S3) and displays the predicted mature protein. All 3D-structures are composed of a α-helix as well as a three-stranded anti-parallel β-sheet. Four disulfide bonds between C1 and C8 are painted in green. The arrow points towards the loop between C5 and C6 of ZmES1, which may be responsible for ZmES specificity. (B) ZmES4 modulates the activity of the potassium channel KZM1. Electrophysiological recordings of ZmES4 action on KZM1 expressed in *Xenopus oocytes*. Typical voltage dependent inward rectifying potassium currents of KZM1 in bath medium with and without control substances such as trypsin inhibitor from soybean or antifungal protein AFP2 from *Raphanus sativus* (left). Loss of voltage dependent gating of KZM1 upon addition of ZmES4 (150 µg/ml) in the bath medium (right). (C) Current voltage relation of KZM1 steady state currents in response to 150 µg/ml ZmES4. Recordings were performed in a bath solution containing 100 mM KCl. Currents were evoked upon voltage jumps in the range of +50 to −180 mV. Data points of four independent experiments were normalized to the value at −160 mV in control solution. The graph on the right depicts a magnification of the data shown on the left in the voltage range between −60 and −100 mV. Due to the loss of voltage dependence in the presence of ZmES4, KZM1 mediates K^+^ currents at all tested membrane potentials. Error bars indicate the standard deviation.

We aimed to identify ZmES4 target(s) and initially analyzed the secondary and tertiary structure of predicted mature ZmES proteins. Based on structure modeling ZmES proteins are more closely related to plant DEFs such as RsAFP2 and invertebrate venom peptides of various species including scorpions, snakes, sea anemones, spiders, insects, marine cone snails, and worms [22]. Less structural homology to other plant CRPs, such as the S-locus group and LURE1/2 (Figures S4 and S5) or the phylogenetic distinct vertebrate DEFs [23], was evident. Despite limited overall sequence identity, ZmES DEFLs and related animal toxins contain a conserved structure consisting of an α-helix and a triple-stranded antiparallel β-sheet (in a βαββ configuration) that is stabilized by 3–4 intramolecular disulfide bonds (Figure 4A) [22]. Although this core structure is highly conserved, the primary sequence displays little amino acid identity. As a consequence, the surface is highly polymorphic (Figure 4A) and different from each other, indicating that these proteins bind to different targets. Animal DEFs/toxins related to ZmES containing the described βαββ-structure have been shown to modulate either K^+^ or Na^+^ ion channels [22] by acting as pore blockers or gating modifiers, supporting the assumption of a role for ion channels in ZmES4-mediated pollen-tube rupture. To test the possibility of CsCl or BaCl_2_ as ion channel blocking agents [24], these ions were added to the pollen-tube growth medium. While addition of 100 µM MgCl_2_ (control) and BaCl_2_ did not show any significant alteration of ZmES4 effects, strong delay of more than eight times of pollen tube rupture was observed when 100 µM of the K^+^ channel specific inhibitor CsCl [24] was present in PGM (Figure 3D). This indicates that K^+^ channels could represent ZmES4 target(s).

To study the activity of potassium channels in response to ZmES4 application we expressed several known channels of the plant shaker K^+^ channel family of maize in *Xenopus* oocytes. This structurally related family consists of three different subgroups: inward rectifying (K^+^ uptake) channels, outward rectifying (K^+^ release) channels, and weakly voltage dependent channels that conduct outward as well as inward K^+^ currents. The rectification properties of these functionally distinct subgroups are based on their voltage dependence. Whereas outward rectifying channels are activating with a time-dependent kinetic upon depolarization, inward rectifying channels are activating with a time-dependent kinetic at hyperpolarized membrane potentials. The third subgroup consists of channels that are only weakly voltage dependent and thus their activation kinetics appear instantaneously. In maize three shaker-like K^+^ channels electrophysiologically characterized are shown to be expressed in pollen tubes (Figure S6). The kinetics of the inward rectifying channel ZMK1 and the weakly voltage-dependent channel ZMK2 [25] remained unchanged by ZmES4 application (Figure S7E). In contrast, the inward rectifying Shaker K^+^ channel KZM1 [26] lost its voltage dependence upon external application of ZmES4 (Figure 4B and 4C). Moreover, KZM1 was inhibited by Cs^+^ in a voltage dependent manner (Figure S7A), supporting the finding that ZmES4 induced pollen tube burst could be significantly delayed by Cs^+^ application. In line with the properties of a voltage-independent channel, KZM1-mediated K^+^ currents appeared with an instantaneous activation kinetic in response to all tested membrane voltages, leading to inward as well as outward K^+^ currents (Figure 3B and 3C, Figure S7B). Interestingly, at membrane potentials more negative than −120 mV the currents in the presence of ZmES4 were smaller than under control conditions. This observation indicates that ZmES4 exhibits a weak inhibitory effect at very negative voltages in addition to its influence on the gating of KZM1. Due to the weak voltage dependence in the presence of ZmES4, the currents reversed at the Nernst potential for potassium. This confirms that the observed ZmES4-induced currents were due to KZM1. Furthermore, the effect of ZmES4 was fully reversible and KZM1 regained voltage dependence and rectification properties upon protein washout (Figure S7B). In contrast to ZmES4, 100 µg/ml of the CRP RsAFP2 [21] changed neither the activation kinetics nor the voltage dependence of KZM1 (Figure S7C and S7D).

## Discussion

After the first description of fertilization in flowering plants more than 120 y ago [4], we are now beginning to understand the underlying molecular and physiological mechanisms involved in pollen tube growth arrest and burst leading to sperm discharge and gamete fusion. Here, we described the activity of the synergid cell secreted DEFL protein ZmES4, required for species-preferential pollen tube burst in maize and its activity on the pollen tube expressed potassium channel KZM1. ZmES4 application triggered membrane depolarization and opening of the pollen tube expressed potassium channel KZM1 at physiological conditions before tube burst, which occurred on average within less than a minute. ZmES4 action is very fast, suggesting that a rapid decrease in the cytosolic water potential represents the main physiological cause of pollen tube burst. Moreover, ZmES4 activity was significantly delayed in the presence of Cs^+^, a K^+^ channel specific blocker, supporting the finding that changes in potassium fluxes play a central role for pollen tube burst in maize. Indeed, osmotic processes represent important forces driving plant growth, movements, and development. Besides sugars, potassium is the major osmotically active solute to maintain plant cell turgor and drives irreversible cell expansion and reversible changes in cell volume [27]. Although the potassium concentration in the filiform apparatus, the entry point of the pollen tube inside the embryo sac, is not known, it was shown that synergid cells contain considerably high potassium concentrations [28]. Once released by the degenerating receptive synergid cell, elevated apoplastic potassium concentrations together with a given hyperpolarized pollen tube membrane potential might provide the necessary driving force allowing rapid potassium influx upon K^+^ channel opening. This hypothesis is further supported by the observation that high cytosolic potassium concentrations in synergid cells are concomitantly lost with pollen tube discharge [28].

In summary we showed that ZmES4-GFP fusion proteins accumulate before fertilization in vesicles at the secretory zone of mature synergid cells, which have been considered to represent the glandular cells of the female gametophyte [29],[30],[31]. Upon pollen tube arrival, vesicles contents seem to be released and disappear within 24 h. Using an RNAi knock-down approach, we found that pollen tubes were guided towards the female gametes but fail to release their contents and occasionally show overgrowth inside the egg apparatus. Using chemically synthesized ZmES4 protein, we further showed that it induced pollen tube rupture in vitro in a species-preferential manner. Moreover, ZmES4 converts the potassium channel KZM1 from a voltage-dependent inward rectifier in a voltage independent, non-rectifying channel. KZM1 is assumed to represent a major housekeeping channel in many tissues and accounts for K^+^ homeostasis. KZM1 has also been considered as a mediator of K^+^ uptake into the phloem as well as guard cells in the leaf epidermis [26]. Pollen tubes have been shown to be especially sensitive to osmotic changes and it is therefore not surprising that a large number of ion channels are involved in osmotic adjustment and K^+^ homeostasis and are expressed during pollen development and tube growth (see also Figure S6) [32]. KZM1 is strongly expressed in pollen tubes, but it cannot be excluded that ZmES proteins also act at other pollen tube expressed targets including Ca^2+^ channels—a property reminiscent to animal toxins capable of modulating various ion channels simultaneously [18],[33]. However, all described activities are capable to lead to a rapid increase in osmotic pressure in the pollen tube, which ultimately results in pollen tube rupture at its weakest point, the very tip lacking callose containing cell wall material. As a consequence, sperm cells and pollen tube tip factors required for fertilization processes are released explosively into the receptive synergid, a process described previously already at the cellular level in *Torenia fournieri* [34]. As described above, this process is species-preferential, as ZmES4 strongly modulated KZM1 activity but obviously showed low activity on ion-channels of related *T. dactyloides* pollen tubes and no significant effect on tubes of other plants tested. We discovered an up to now unknown additional species-specific reproductive hybridization barrier that could represent a further component of reproductive isolation and, thus, speciation in plants [29],[35],[36]. Moreover, this finding opens the possibility to introduce ZmES related CRPs of the DEFL subclass in embryo sac cells or potassium channels in pollen tubes of various grass species to overcome species-specific crossing barriers.

The surface loop that is responsible for species-specific antifungal activity of the plant DEF RsAFP2 has been mapped as C5-C6-loop [21],[37], the same loop that protrudes from the ZmES1 structural model (arrow in Figure 4A). This region differs in ZmES1 and ZmES2-4 indicating that ZmES CRPs may indeed target different proteins. Additionally, this region indicates a first sub-domain that could be engineered in order to overcome its species-specific properties.

Considering that the egg apparatus secretes a large cocktail of diverse small CRPs [11],[12],[13],[14],[15],[16], perhaps more ion channels and/or receptors are affected simultaneously culminating in explosive tube discharge. This hypothesis is supported by the finding that RNAi silencing of the whole *ZmES* family did not lead to the expected 50% reduction of the fertilization rate. However, it also cannot be ruled out that the knock-down effect was indeed 100% efficient. Thus a systematic approach is now required to analyze the functions and activities of the various embryo sac expressed *DEFL* genes on all pollen tube expressed ion channels and other receptors. Interestingly, our findings show that pollen tube growth inhibition and discharge is not only mechanistically related to defense mechanisms against fungal attack, but moreover similar molecular players are involved. While many DEFs are required to protect both plant and animal reproductive tissues from pathogens [29],[38],[39], ZmES proteins obviously evolved a novel function, although still possessing low antifungal activity. As indicated above, a large number of *DEFL* genes are also expressed in the *Arabidopsis* and *Torenia fournieri* female gametophyte [11],[15],[20] indicating that the reported physiological mechanism of tube burst might well be conserved in angiosperms and that more *DEFL* genes obtained other functions. Hoverer, due to the polymorphic nature of *DEFL* genes, orthologous genes cannot be predicted and have to be identified experimentally. For example, related *DEFL* genes *LURE1* and *LURE2* from *Torenia fournieri* have recently been shown to encode the female gametophyte secreted attractants of the pollen tube [20] providing further support for the assumption that *DEFL* genes have evolved specific functions during plant reproduction. Interestingly, a similar evolutionary phenomenon has recently been proposed for the FER RLK: a homozygous *fer* mutant in *Arabidopsis* leads to fungal resistance [40], although the function of this RLK in the ovule seems to be restricted to pollen tube growth arrest [5]. Thus, after the identification of the first players involved in pollen tube growth arrest and burst, it will now be exciting to find out how the pathways are connected and how they evolved: for example, do FER or LRE [6] signaling pathways lead to secretion of ZmES1-4 containing vesicles similar to the release of cortical granules of animal species [41]? Do ZmES proteins also act on maize homologs of *Arabidopsis* ACA9 Ca^2+^ pumps [9] whose activity is also required for pollen tube burst? And finally, can we switch back the evolutionary clock and engineer “reproductive” DEFLs into “defense” DEFLs and vice versa? Systematic approaches to study female gametophyte expressed *DEFL* gene families will provide exciting answers during the next few years.

## Materials and Methods

### Plant Material and Growth

Maize inbred lines A188 and H99 as well as transgenic lines were grown under standard greenhouse conditions at 26°C with 16 h light and a relative air humidity of about 60%. Pollen grains were harvested as follows: ears were shaken in the early morning to remove old pollen grains. At 9–10 a.m. tassels containing mature pollen grains were covered by paper bags and hand shaken to collect fresh pollen that was used to pollinate ears, which were bagged before silk emergence. Silks were cut to 4–8 cm immediately before pollination. Arabidopsis seeds were vernalised in growth chambers for 2 d at 4°C without light and an average air humidity of about 55%–60%. After vernalisation seeds were germinated and grown in growth chambers at long day conditions with 16 h light (22°C and 20°C in the dark). *Pseudomonas syringae* inoculated Arabidopsis plants were grown in separate growth chambers under short day conditions with 9 h of light (18°C). Onion bulbs for transient transformation were obtained from AGRATA GmbH.

### Generation of Constructs, Biolistic Transformation, and Generation of Transgenic Plants

To generate the *ZmES4-GFP*-fusion construct (*ZmES4*p*:ZmES4-GFP*) for maize transformation, 1,620 bp upstream of the *ZmES4* ORF was used as the promoter region. The *eGFP* gene was PCR amplified from the eGFP expression vector pMon30049 [42] with the primers GFPBam (5′-GGATCCGGCCGATGGGCAAGGGC-3′) and GFPXho (5′-CTCGAGTCACTTGTAGAGTTCATCC-3′) and digested with *Bam*HI and *Xho*I. The NOS-terminator was amplified from pBi121 with primers SANF (5-GTCGACTCGAATTTCCCCGATC-3′) and NOEco (5′-GAATTCCCGATCTAGTAAC-3′) and digested accordingly. The *ZmES4* promoter was amplified from maize inbred line A188 genomic DNA with primers PESSpe (5′-ATAGTTATTGATCTACTGGTCATGTAC-3′) and PESr (5′-CTGTGTCAGGCAGTC-3′). The *ZmES4* ORF was amplified from cDNA clone ZmEC44/6[12] with primers CESf (5′-GTTCCACCACATTACTTCC-3′) and ESBsp (5′-GCGACTAGTTCCACCACATTAC-3′). The resulting fragments were amplified through overlapping PCR with primers PESSpe (5-ACTAGTTATTGATCTACTGGTCATGTAC-3) and ESBsp (5-GGGCCCTTTTGTCGTGGTGGATGTGC-3), restricted with *Spe*I and *Bsp*120, and cloned into the appropriate interfaces of the vector L29GFPeag (DNA Cloning Service). For transient expression studies, the *ZmES4* promoter was replaced by the maize *ubiquitin* promoter present in the vector pLNU-GFP (DNA Cloning Service). The ORF of *ZmES4* was amplified from clone ZmEC44/6 [12] with primers ESR-GFP (5′-GTCGGATCCATTTTGTCGTGGTG-3′) and ESF-GFP (5′-CGCGACTAGTTCCACCACATTAC-3′). The resulting fragment was cloned into the corresponding restriction sites (*Spe*I-*Bam*HI) of the pLNU-GFP vector. This vector was also used as a positive control for transient biolistic transformation experiments.

Epidermal onion cell layers were bombarded with 2–5 µg plasmid DNA according to the procedure described [43], except that inner onion peels (2×2.5 cm) were placed with the concave side up on 0.5% agar plates. The condition of bombardment was 1,100 psi rupture discs under a vacuum of 28 mm Hg with 6 cm target distance using the Particle gun model PDS100/He (BioRad). Bombarded peel-halves were placed after transformation with the concave side down and the cut surface in sterile 0.6% agar Petri dishes for about 17–22 h in the dark before removing the epidermis for observation using a fluorescence microscope.

For stable transformation of immature maize embryos, 5 µg plasmid DNA was precipitated onto gold particles with an average size of 0.4–1.2 mm (Heraeus) following a modified protocol [44] originally described by BioRad. Particle-DNA pellets were first resuspended in 240 µl ethanol and then 3.5 µl was each spread on the macrocarrier for particle bombardment. Immature embryos were isolated 12–13 d after pollination from A188 inbred ears after pollination with pollen from maize inbred line H99. Isolated hybrid embryos were pre-cultured 8–14 d and an additional 4 d in osmotic medium before bombardment. Co-transformation was carried out with a *35S*p:*PAT*-construct carrying the selectable marker PAT for glufosinate ammonium resistance (to select for BASTA® resistance). Particle bombardment, tissue culture, and selection of transgenic maize plants were carried out as described [45]. To study the role of *ZmES* genes during fertilization, an RNAi construct was generated to silence the whole gene-family simultaneously. The construct was designed as follows: 400 bp of the clone *ZmEC 44/6* was amplified in sense orientation with primers ES4F-Eco (5′- GTCAGAATTCACCACATTACTTCCA-3′) and ES4R-Bam (5′-GACTGGATCCCAAGACATTTACAA-3′) as well as inverse with primers ES4F-Bsr (5′- GCGCTGTACACCACATTACTTCCA-3′) and ES4R-Mlu (5′-GACTACGCGTCCCAAGACATTTACAA-3′). After digestion with respective restriction enzymes, both fragments were cloned into the corresponding splicing sites of the *pUbi-iF2* vector that contains a maize *Ubi* promoter, 1,121 bp of the *Fad2* intron, and the *NOS* terminator. This construct was generated and like the other constructs sequenced by DNA Cloning Service.

To constitutively express *ZmES4* in *Arabidopsis* for pathogenity tests, the vector pBD515.3 [46] as well as the clone *ZmEC 44/6* carrying a *ZmES4* cDNA were cut with *Bam*HI and *Kpn*I, ligated, and fully sequenced with the primer ZmES5AF (5′-AAAACGAATAATAATCCGGCAATGGAGTCTT-3′). Arabidopsis shoot apical meristems were transformed via the Agrobacterium-mediated vacuum infiltration method previously described [47].

### Analyses of Transgene Integration and Expression Studies

Genomic DNA from maize leaves was isolated based on the described method [48] with some modifications. 200–300 mg leaf material was frozen in liquid nitrogen and ground in 2 ml Eppendorf tubes containing two small steel balls using the Retsch swinging mill MM 2000 (Retsch). Extraction buffer was added to pulverised leaf material and extracted twice with phenol. After centrifugation Na_2_Ac (3 M, pH 5.2) was added to the aqueous part and precipitated with Isopropanol. Finally, the pellet was washed twice with EtOH (80%) and digested in R40 (10 mm Tris-HCl: pH 8.0, 1 mm EDTA: pH 8.0 and 40 µg/ml RNAse A) for 3–6 h at 37°C. Isolation of genomic DNA from *Arabidopsis thaliana* was done based on the method described [49]. Capillary Southern and Northern blots as well as labeling, hybridization, washing, and autoradiographic exposures were performed as described [50]. Probes to detect *ZmES-GFP* transgene integrations were isolated from plasmids that have been used for maize transformations. DNA probes for radiographic detection were generated as described [50] or performed with Digoxigenin-11-dUTP (DIG) by PCR according to protocols provided by Roche Molecular Biochemicals. A *GFP*-specific probe was prepared as follows: the *GFP* sequence was amplified in a standard PCR using the primers NOS (5′-CAAGACCGGCAACAGGATTC-3′) and GFPF (5′-GACTATCTTCTTCAAGGATGAC-3′). A *ZmES4*-specific probe was prepared using the primers 200a (5′-CCCTTGGATTGGATTGGATCG-3′) and 200b (5′-GAAGTCTGTGGTG3′).

In order to study the expression of various potassium channels in germinated pollen tubes of maize, fresh pollen of the maize inbred line A188 was germinated in 30 drops of each 5 µl PGM (pollen germination medium [51]) for 40 min. Drops were transferred into a 1.5 ml centrifuge tube and collected by centrifugation at 100× g for 60 s and immediately frozen in liquid nitrogen. For cDNA synthesis mRNA was isolated using the Dynabeads® mRNA DIRECT™ Micro kit (Dynal® Biotech, Invitrogen) according to the manufacturers' instructions using 2-fold lysis-binding buffer. cDNA was synthesized using Transcriptor High Fidelity cDNA Synthesis Kit (Roche). mRNA annealed to magnetic Dynabeads was transferred using a PickPen™ (Bio-Nobile). Quality and amount of generated cDNAs were checked by PCR using intron-flanking *GAPDH-*specific primers GAPDHfor (5'-AGGGTGGTGCCAAGAAGGTTG-3') and GAPDHrev (5'-GTAGCCCCACTCGTTGTCGTA-3') as well as *Actin 81/83*-specific primers ZmAct 81/83fw (5'-GGTGATGGTGTGTCT-3') and ZmAct 81/83rev (5'-ACTGAGCACAATGTTAC-3'). Expression of four known maize K^+^-channels was analyzed using the following primer pairs: KZM1-fw3 (5'-CGAGCTATTCCATGCTCCTC-3') and KZM1-rv3 (5'-GGTCATGGCTGTTTCCTTGT-3') for KZM1, KZM2-LC-fw3 (5'-TCATGTATATCCACAGCAGAAC-3′) and KZM2-LC-re3 (5′-TGATGATATTGAGCTTCCGT-3') for KZM2, ZMK1-LC-fw (5'-ATAACAATGGGCATACAG-3') and ZMK1-LC-re (5'-TTCCGTCTTTCATTGAG-3') for ZMK1, as well as ZMK2-LC-fw (5'-TCCTCAGAAACCGCAC-3') and ZMK2-LC-re (5'-CGATCAACGCCTTCTC-3') for ZMK2. Gene bank accession numbers are indicated in the legend of Figure S6. RT-PCR was performed using SYBR® Advantage® qPCR Premix (Clontech) in an Eppendorf Mastercycler® ep *realplex* with initial denaturation for 45 s at 95°C and 40 cycles of amplification with 8 s denaturation at 95°C, 20 s of annealing at 59°C, and 15 s of extension at 72°C followed by melting curve analysis performed from 55–95°C.

### Fluorescence Microscopy

With the exception of the bioassay experiments described below, Axiovert 35 M or Axiovert 200 inverted fluorescence microscopes (both Zeiss) with the filter set 01 (FITC filter with excitation at 450–490 nm; emission at >515 nm) or filter set 38 (GFP filter with excitation at 470–495 nm; emission at 525 nm) were used to observe GFP fluorescence in onion epidermal and maize ovule sections. A DAPI filter (Zeiss, excitation at 359–371 nm and emission >397 nm) was used to visualize DNA and cell wall material. Samples were excited with UV-light produced by a HBO 50/Ac lamp and images taken with a Nikon DS-5Mc camera (Nikon). Nikon software EclipseNet was used to obtain and merge fluorescence images. Confocal laser scanning microscopy was performed using the Leica TCS 4D CLSM (Leica-Laser-Technologie, Heidelberg, Germany). Samples were excited by 488 nm with an Argon laser and monitored as described [52].

### In Vitro Pollination and Histochemical GUS Assay

In vitro pollination was performed as described earlier [12] with some modifications. Unfertilized maize ears (silk length of 5–10 cm) were harvested and, after removing all hull leaves, first cut longitudinally and then transversally to obtain segments of about 5 to 6 cm length. Segments were kept on wet lab paper in a Petri dish (15 cm in diameter) with the ovule containing part of the ear upwards. All silks were orientated towards one direction, cut to the same length, and pollinated with freshly harvested *ACT*p:*GUS* pollen. To avoid drying, segments were covered within the Petri dish with wet filter paper. Sections through maize ovules were isolated from ovaries 27–32 hap as well as 53–55 hap, which corresponds to ∼22 h and ∼46 h after fertilization, respectively. Further on, these sections were used for histochemical GUS-assays and have been incubated overnight at 37°C in staining buffer containing X-Gluc (5-bromo-4-chloro-3-indolyl-β-D-glucuronic acid CHA-salt) as described [17]. Stained samples were analyzed by light microscopy using an Axiovert 200 microscope (Zeiss). Documentation was done using a CAMEDIA C-4040 ZOOM digital camera (Olympus), and images were processed with Adobe Photoshop CS2 (Adobe Systems Inc.).

### In Vitro Pollination and ZmES4 Bioassay

The mature predicted ZmES4 microprotein (61 amino acids) was chemically synthesized with 80%–-90% HPLC-purity by JPT Peptide Technologies GmbH. Successful synthesis of the linear peptide was shown by LC-MS. Intra-molecular disulfide-bridges were introduced via thermodynamically controlled folding. 0.5 mg of the ZmES4 protein was each first dissolved in DMSO and then in double distilled water. Double concentrated PGM [53] was added with a final concentration of DMSO not exceeding 2% and ZmES4 microprotein available in 1× PGM. Trypsin inhibitor from soybean (Sigma), RsAFP2, and LURE2 were each dissolved using the same method described above.

Fresh pollen grains of maize and *Tripsacum dactyloides* were shaken directly onto 10 µl drops of 1× PGM placed in a 35 m m plastic Petri dish. Pollen of tobacco (*Nicotiana benthamiana* and *Nicotiana tabaccum*) and lily (*Hemerocallis fulva*) was transferred directly from a fresh opened anther onto drops of 1× PGM using forceps. Germination was performed at room temperature for 20 to 120 min, depending on plant species analyzed. Germinated pollen tubes were subjected to biological assays only if germination rates exceeded 75%. During this assay, droplets were observed using an Eclipse TE2000 inverted microscope (Nikon) equipped with a 1.4 Megapixel digital AxioCam MRm camera and AxioVision digital image processing software (both Zeiss).

A 10 µl solution either of ZmES4/PGM or control CRPs/PGM was added to each droplet with germinated pollen using the CellTram® Air (Eppendorf) and a glass micropipette. A time series was started directly after protein addition and mixing, and pollen burst recorded for up to 120 min or stopped earlier when pollen tube burst was completed. In order to block potassium, sodium, or other cation channels, which may be targets of the ZmES4, pollen was germinated in 10 µl droplets of PGM for 15 min containing either 200 µM CsCl, MgCl_2_, BaCl_2_, GdCl_3_, or LaCl_3_. Then 10 µl ZmES4/PGM solution was added to the droplet and the number of bursted pollen tubes was determined as described above.

### Electrophysiological Experiments

A functional analysis of maize K^+^ channels was performed by two-electrode voltage clamp technique following heterologous expression in *Xenopus* oocytes. Generation of cRNA and preparation of oocytes have been described previously [54]. Two-electrode voltage-clamp recordings were performed making use of a TURBO TEC 3× amplifier (NPI Electronic GmbH) integrated in an automated perfusion system developed for fast and efficient application of small amounts of drugs/solutions (ScreeningTool, NPI Electronic GmbH [55]). Voltage clamp recordings were performed in external solution containing 1 mM CaCl_2_, 1 mM MgCl_2_, 10 mM Tris-MES pH 7.4, 100 mM KCl, and 2% (w/v) DMSO. ZmES4 was applied at a final concentration of 150 µg/ml. Currents were elicited by voltage jumps in the range of −180 to +60 mV (10 mV increments) starting from a holding potential of −10 mV. To study the inhibition by the K^+^ channel specific inhibitor Cs^+^ [24], 5 mM CsCl was added to the standard solution. Following an activating voltage pulse of −130 mV, tail currents (I_t_) at *t*  = 0 were extracted from test pulses in the range of −180 to +60 mV (10 mV increments) and plotted against the applied voltage.

Impalement of pollen tube tips growing on ½ MS agar: growing pollen tubes were impaled right below the tip, using a micromanipulator (type 5171; Eppendorf) in combination with a piezo translator (P-280.30, Physik Instrumente). Micro-electrodes were pulled from borosilicate capillaries (OD 1 mm, i.d. 0.58 mm, with filament; Hilgenberg), filled with 300 mM KCl and connected by an Ag/AgCl half cell to the microelectrode amplifier (VF-102; Bio-Logic). Current-clamp measurements were recorded with PULSE software (HEKA Electronics). Data were low pass filtered at 300 Hz (8-pole Bessel-filter type 902; Frequency Devices) and sampled at 1 kHz. ZmES4 or control CRP containing liquid PGM was applied by a Drummond Nanoliter Injector (WPI).

### Phytopathology Tests

Bacterial inoculations: 4- to 5-wk-old *Arabidopsis* plants were hand infiltrated with *Pseudomonas syringae* pv. tomato DC3000 suspensions with 10^5^ cfu/ml. At different time points (0, 1, 3, and 5 days after inoculation [DAI]) bacterial growth was analyzed by macerating two leaf discs per replicate in 10 mM MgCl_2_ and plating serial dilutions of the bacterial suspension on Kings B medium supplemented with 50 mg/l Rifampicin and 50 mg/l Cycloheximide. Assessment of *Peronospora parasitica* sporulation: different *Arabidopsis* ecotypes were sown onto a mixture of three parts of seedling compost and one part of sand in aluminum test tube caps with a diameter of 2.8 cm. After stratification for 2 d at 4°C, seedlings were further cultivated at 18°C in a growth cabinet with a 9 h day/15 h night cycle. 7- to 8-d-old seedlings were spray inoculated with a conidial suspension of the *P. parasitica* isolate Emwa 1 [56] at concentrations of either 10^3^ or 10^5^ conidia/ml in distilled water. The seedlings were placed in a tray covered with a clear plastic lid to maintain approximately 100% relative humidity. The degree of colonization was monitored 10, 30, and 70 DAI. For the latter time point plantlets were picked individually into 9 cm pots containing potting compost and sand (3:1).

### Bioinformatics

ZmES1-4 sequence data (Genbank accessions NP_001104950, NP_001106003, NP_001106035, and NP_001105620) were compiled and compared online with GenBank, SwissProt, PIR, and PRF databases with default BLAST algorithms [57] and aligned online by ClustalW (http://www.ebi.ac.uk/clustalw/) [58]. Protein alignments were drawn by GeneDoc version 2.6.02 [59]. Prediction of protein localization and processing sites was performed online using PSORT (http://psort.nibb.ac.jp), iPSORT (http://www.HypothesisCreator.net/iPSORT), and SignalP V2.0 (http://www.cbs.dtu.dk/services/SignalP-2.0). 3D-modeling was performed using Discovery Studio 1.5 software (Accelrys Limited) and constructs were generated and drawn using Clone Manager 6 (Sci-Ed Software).

## Supporting Information

Figure S1 **ZmES4 protein is secreted via the secretory pathway.** (A–M) Onion cells were transiently transformed with a *UBI*p:*ZmES4-GFP* construct and analyzed using epifluorescence combined with bright field microscopy (A and C) or epifluorescence alone (B, D–M). (A) Fusion protein accumulates around the nucleus (arrowhead) and within large vesicles probably representing golgi stacks (arrows). The asterisks mark cell walls of neighboring cells displaying weak GFP signals. (B) The same image as in (A), but signals in cell walls of neighboring cells are more clearly visible. Encircled is a number of secretory vesicles close to the plasma membrane. (C) Control showing an onion epidermal cell bombarded with a *35S*p:*Lc-GFP* construct encoding the N-terminal 388 aa (incl. the NLS) of a maize transcriptional regulator of anthocyanin biosynthesis in maize (GenBank accession #A41388) fused with GFP. Most of the fluorescence is visible within the nucleus (arrowhead). (D) Epifluorescence of the image shown in (C). (E–M) Time course displaying movement of a secretory vesicle and fusion with the plasma membrane. The start point of the vesicle is indicated by an arrow and the time interval from the first image is given in the bottom left corner of the images. Fusion is visible from image (I) onwards (indicated by two small arrowheads). (N and O) CLSM sections through the micropylar region of the egg apparatus as in Figure 1D. A few vesicles are labeled by asterisks.
(1.16 MB PDF)

Figure S2 **Overexpression of ZmES4 in** ***Arabidopsis thaliana*** **to study its role as a defensin.** (A) Leaves of the susceptible ecotype Wassilewskija (WS) were infected with the bacterial pathogen *Pseudomonas syringae* pv. tomato DC3000 (Pst). The image shows phenotypes of infected leaves from wild type control [wt (+)] plants, leaves from overexpressing plants [*35S*:*ZmES4* (+)], and non-infected control plants [wt(−)] 7 DAI. (B) Quantification of bacterial growth at 1, 3, and 5 DAI in leaves of susceptible wild type (wt) and overexpressing (35S:ZmES4) plants. Bars represent mean value of 20 to 39 leaf samples analyzed per stage and small bars indicate standard deviations. (C) Seedlings were inoculated with the fungal pathogen *Peronospora parasitica.* 10 DAI, seedlings infected with 10^3^ spores/ml of the susceptible ecotype WS and of overexpressing plants displayed hyphae and conidiophores, while resistant ecotype Landsberg erecta (Ler) did not show visible fungal growth. (D) 40 DAI at high spore concentration of 10^5^ spores/ml; overexpressing plants (top row) were more vital, started flowering, and fungal growth was no longer visible. In contrast, susceptible wild type plants (bottom row) grew smaller and hyphae and conidiophores were still visible at older leaves.
(0.25 MB PDF)

Figure S3 **ZmES4 concentration dependent induction of pollen tube burst.** Percentage of maize pollen tube burst was measured 2 min after application of 30 nM up to 30 µM ZmES4. 7–11 experiments with a total of up to 300 pollen tubes for each experimental conditions have been recorded. Average numbers of pollen tube burst are given. Neither 30 µM RsAFP2 nor LURE2 (not shown) did induce pollen tube burst, while 1,000 times lower concentrations of ZmES4 still induces burst of 1/3 pollen tubes. PGM (pollen germination medium) was used as a negative control.
(0.07 MB PDF)

Figure S4 **Alignment of the predicted mature ZmES1 protein and structural related proteins.** ZmES proteins represent a novel knottin-subclass of cysteine-rich microproteins (CRPs) with structural similarity to neurotoxins and animal and plant defensins, and less homology to the male determinant of *Brassica* self-incompatibility or the pollen tube attractant LURE2 (TfCRP3). Structural comparison of the predicted mature ZmES1 and ZmES4 proteins with predicted mature proteins of the large gene-family of *Arabidopsis thaliana* low-molecular-weight cysteine-rich (LCR) proteins (AtLCR72: At2g02140), antifungal protein of *Raphanus sativus* (RsAFP2: P30230), TfCRP3 (BAH29751) of *Torenia fournieri*, two variants of the *Brassica rapa* highly polymorphic S-locus cysteine-rich protein 11 (S_8_-SP11: BAA92246 and S_9_-SP11: BAA85458), as well as the pollen coat protein PCP1 (BAA25682), the Sahara scorpion (*Androctonus australis*) neurotoxin II (AaToxII: 1PTX), the Egyptian scorpion *Leiurus quinquestriatus* insect toxins 1 (LqIT1: P19856), insect defensin A (PtIDEFA: 1ICA) from flesh fly *Protophormia terraenovae*, defensin I (AmDEFI: P17722) of honey bee *Apis mellifera*, as well as antimicrobial Sapecin-B (SpSAPB: P31529) from flesh fly *Sarcophaga peregrine*. Please note that the last three proteins and TfCRP3 are predicted to form three intramolecular disulfide bonds, while the other proteins form four bonds between cysteine residues C1–C8 as indicated. The length of predicted mature peptides is indicated at the right.
(0.21 MB PDF)

Figure S5 **Alignment of ZmES1 and ZmES4 precursors and close plant homologs.** Close plant homologs; maize ZmLCR1: AY112374, ZmLCR2: AY108650, and ZmLCR3: AY105007, rice: OsLCR1: Os02g41910, OsLCR2: AL662939, and OsLCR3: Os03g03810; *Arabidopsis thaliana*: AtLCR69: At2g02100, AtLCR71: At2g02135, AtLCR72: At2g02140, and AtLCR74: At5g63660; spruce: PgLCR1: AAR84643 of *Picea glauca* and PaSPI1B: AAN40688 of *Picea abies*. Predicted N-terminal signal sequences are boxed. Eight conserved cysteine residues C1–C8 are highlighted in black, acidic amino acids in red, and basic amino acids in blue. Red lettering displays hydrophobic amino acids, aromatic amino acids are shown in pink, and serine as well as threonine in dark green. A consensus sequence of this knottin/defensin-subclass of cysteine-rich microproteins is given below mature protein sequences. Numbers at the right display length of precursor sequences.
(0.40 MB PDF)

Figure S6 **Expression analysis of potassium channels in pollen tubes of the maize inbred line A188.** The expression of four known maize potassium channels was studied by RT-PCR using cDNA from pollen tubes as described in Supporting Information. KZM1 (AJ421640) & KZM2(AJ558238): K^+^ channels *Zea mays* 1 and 2; ZMK1 (Y07632) & ZMK2 (AJ132686): *Zea mays* K^+^ channel 1 and 2; RT-PCR controls: ACT: actin 81/83 (AAB40105); GAPDH (X07156): glycerinaldehyde 3-phosphate dehydrogenase. **c:** cDNA & **g:** genomic DNA was each used as a template, respectively. The size of various genomic PCR products (KZM2, ZMK1, ZMK2, and GAPDH) is larger than that of cDNAs indicating that the cDNA used as a template did not contain genomic DNA. M: 100 bp DNA ladder was used to visualize the length of the amplified DNA fragments.
(0.21 MB PDF)

Figure S7 **Reversibility of KZM1 activation by ZmES4 and inhibition of KZM1 currents by Cs^+^.** (A) Instantaneous potassium currents (I_t_) elicited by oocytes expressing KZM1 were inhibited by 5 mM Cs^+^ in a voltage dependent manner. Recordings were performed at membrane potentials in the range of +60 to −180 mV in a bath solution containing 95 mM KCl at pH 7.4. Data points of four independent experiments were normalized to the value at −160 mV in control solution. Error bars indicate the standard deviation. (B and C) Whole oocyte currents recorded in 100 mM KCl in the presence and absence of ZmES4 (B) or RsAFP2 (APP in C). Starting from a holding potential of −10 mV a single voltage pulse to −100 mV was applied. Due to the loss of voltage dependence, the application of ZmES4 altered the activation kinetics of KZM1 from a time-dependent activation to a instantaneous activation. Reversion to voltage dependence and time-dependent activation kinetics of KZM1 is observed after washout of ZmES4. In contrast RsAFP2 did not alter the activation kinetics of KZM1. (D) Current voltage relation of KZM1 steady state currents in response to 100 µg/ml RsAFP2. Recordings were performed as follows: currents were evoked upon voltage jumps in the range of +20 to −150 mV in a bath solution containing 100 mM KCl. Data points of four independent experiments were normalized to the value at −150 mV in control solution. Error bars indicate the standard deviation. (E) Application of ZmES4 did not affect kinetics of the maize potassium channels ZMK1 (left panel) or ZMK2 (right panel). Recordings were performed in 100 mM KCl, pH 7.4. Currents were monitored in response to voltage changes ranging from +20 to −170 mV in 10 mV decrements.
(0.21 MB PDF)

Table S1 **Comparison of fertilization rates after in vitro pollination of WT and ZmES4-RNAi ovules using GUS-labeled pollen tubes.**
(0.06 MB DOC)

## Abbreviations

CRPs: cysteine-rich proteins
DAI: days after inoculation
DEF: defensin
DEFL: defensin-like protein
ER: endoplasmic reticulum
GFP: green fluorescent protein
hap: hours after pollination
KZM1: K^+^ channel *Zea mays* 1
LTPs: lipid transfer proteins
MYA: million years ago
RALF: rapid alkanization factor
RLK: receptor-like kinase
ZmEA1: *Zea mays* egg apparatus 1
ZmES4: *Zea mays* embryo sac 4
ZMK1/2: *Zea mays* K^+^ channels 1/2
