# Supplementary figures and images for: Defensin-Like ZmES4 Mediates Pollen Tube Burst in Maize via Opening of the Potassium Channel KZM1
Source: PLoS Biol. 2010 Jun 1;8(6):e1000388. doi: 10.1371/journal.pbio.1000388 (PMC2879413; doi:10.1371/journal.pbio.1000388)

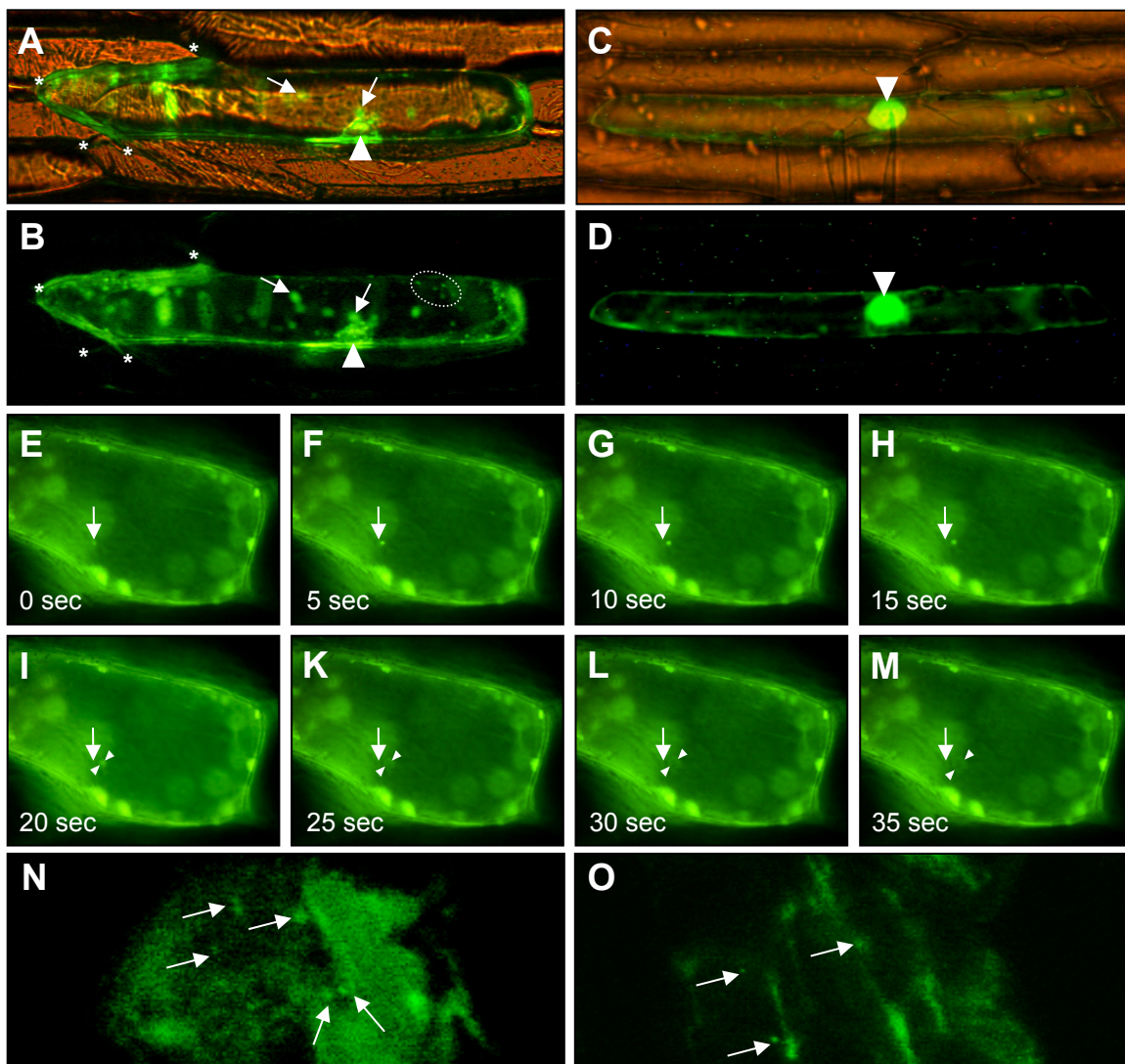

Supplement: Figure S1 — ZmES4 protein is secreted via the secretory pathway. (A–M) Onion cells were transiently transformed with a UBIp:ZmES4-GFP construct and analyzed using epifluorescence combined with bright field microscopy (A and C) or epifluorescence alone (B, D–M). (A) Fusion protein accumulates around the nucleus (arrowhead) and within large vesicles probably representing golgi stacks (arrows). The asterisks mark cell walls of neighboring cells displaying weak GFP signals. (B) The same image as in (A), but signals in cell walls of neighboring cells are more clearly visible. Encircled is a number of secretory vesicles close to the plasma membrane. (C) Control showing an onion epidermal cell bombarded with a 35Sp:Lc-GFP construct encoding the N-terminal 388 aa (incl. the NLS) of a maize transcriptional regulator of anthocyanin biosynthesis in maize (GenBank accession #A41388) fused with GFP. Most of the fluorescence is visible within the nucleus (arrowhead). (D) Epifluorescence of the image shown in (C). (E–M) Time course displaying movement of a secretory vesicle and fusion with the plasma membrane. The start point of the vesicle is indicated by an arrow and the time interval from the first image is given in the bottom left corner of the images. Fusion is visible from image (I) onwards (indicated by two small arrowheads). (N and O) CLSM sections through the micropylar region of the egg apparatus as in Figure 1D. A few vesicles are labeled by asterisks. (1.16 MB PDF) [file pbio.1000388.s001.pdf]

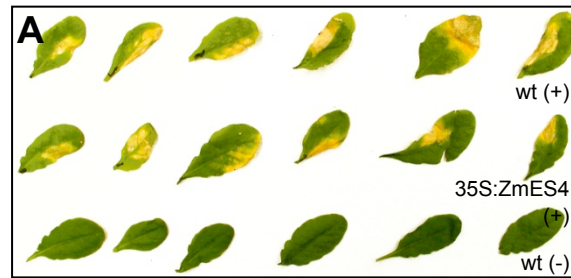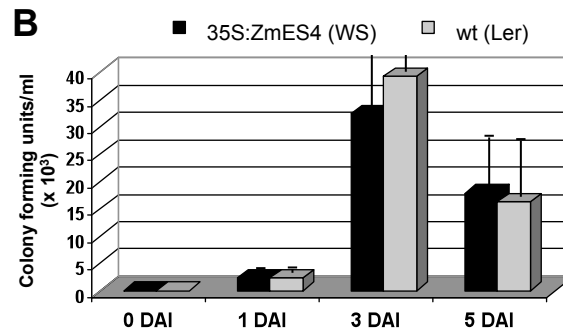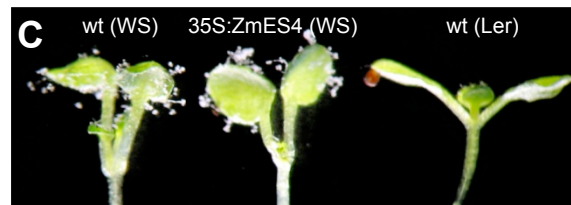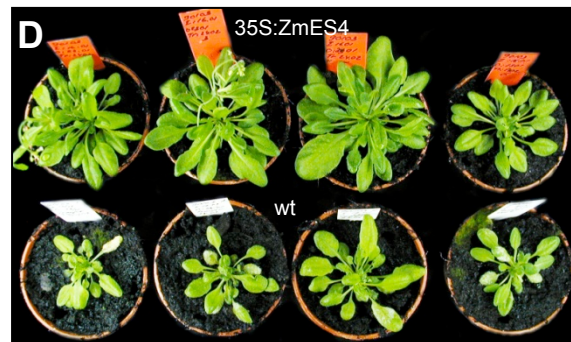

Supplement: Figure S2 — Overexpression of ZmES4 in Arabidopsis thaliana to study its role as a defensin. (A) Leaves of the susceptible ecotype Wassilewskija (WS) were infected with the bacterial pathogen Pseudomonas syringae pv. tomato DC3000 (Pst). The image shows phenotypes of infected leaves from wild type control [wt (+)] plants, leaves from overexpressing plants [35S:ZmES4 (+)], and non-infected control plants [wt(−)] 7 DAI. (B) Quantification of bacterial growth at 1, 3, and 5 DAI in leaves of susceptible wild type (wt) and overexpressing (35S:ZmES4) plants. Bars represent mean value of 20 to 39 leaf samples analyzed per stage and small bars indicate standard deviations. (C) Seedlings were inoculated with the fungal pathogen Peronospora parasitica. 10 DAI, seedlings infected with 103 spores/ml of the susceptible ecotype WS and of overexpressing plants displayed hyphae and conidiophores, while resistant ecotype Landsberg erecta (Ler) did not show visible fungal growth. (D) 40 DAI at high spore concentration of 105 spores/ml; overexpressing plants (top row) were more vital, started flowering, and fungal growth was no longer visible. In contrast, susceptible wild type plants (bottom row) grew smaller and hyphae and conidiophores were still visible at older leaves. (0.25 MB PDF) [file pbio.1000388.s002.pdf]

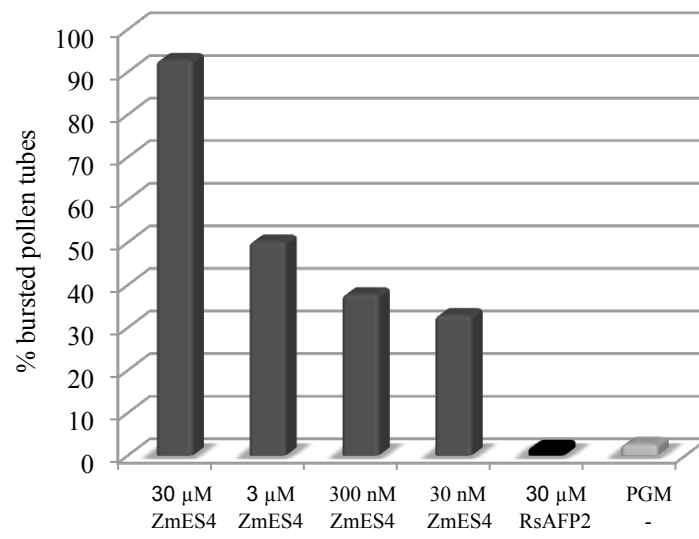

Supplement: Figure S3 — ZmES4 concentration dependent induction of pollen tube burst. Percentage of maize pollen tube burst was measured 2 min after application of 30 nM up to 30 µM ZmES4. 7–11 experiments with a total of up to 300 pollen tubes for each experimental conditions have been recorded. Average numbers of pollen tube burst are given. Neither 30 µM RsAFP2 nor LURE2 (not shown) did induce pollen tube burst, while 1,000 times lower concentrations of ZmES4 still induces burst of 1/3 pollen tubes. PGM (pollen germination medium) was used as a negative control. (0.07 MB PDF) [file pbio.1000388.s003.pdf]

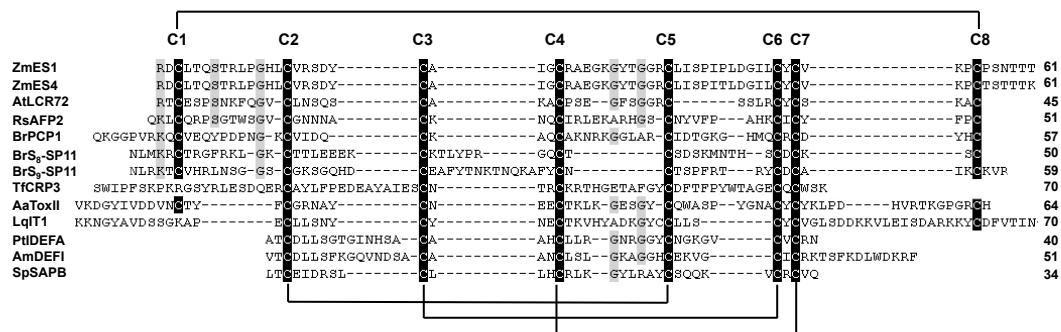

Supplement: Figure S4 — Alignment of the predicted mature ZmES1 protein and structural related proteins. ZmES proteins represent a novel knottin-subclass of cysteine-rich microproteins (CRPs) with structural similarity to neurotoxins and animal and plant defensins, and less homology to the male determinant of Brassica self-incompatibility or the pollen tube attractant LURE2 (TfCRP3). Structural comparison of the predicted mature ZmES1 and ZmES4 proteins with predicted mature proteins of the large gene-family of Arabidopsis thaliana low-molecular-weight cysteine-rich (LCR) proteins (AtLCR72: At2g02140), antifungal protein of Raphanus sativus (RsAFP2: P30230), TfCRP3 (BAH29751) of Torenia fournieri, two variants of the Brassica rapa highly polymorphic S-locus cysteine-rich protein 11 (S8-SP11: BAA92246 and S9-SP11: BAA85458), as well as the pollen coat protein PCP1 (BAA25682), the Sahara scorpion (Androctonus australis) neurotoxin II (AaToxII: 1PTX), the Egyptian scorpion Leiurus quinquestriatus insect toxins 1 (LqIT1: P19856), insect defensin A (PtIDEFA: 1ICA) from flesh fly Protophormia terraenovae, defensin I (AmDEFI: P17722) of honey bee Apis mellifera, as well as antimicrobial Sapecin-B (SpSAPB: P31529) from flesh fly Sarcophaga peregrine. Please note that the last three proteins and TfCRP3 are predicted to form three intramolecular disulfide bonds, while the other proteins form four bonds between cysteine residues C1–C8 as indicated. The length of predicted mature peptides is indicated at the right. (0.21 MB PDF) [file pbio.1000388.s004.pdf]

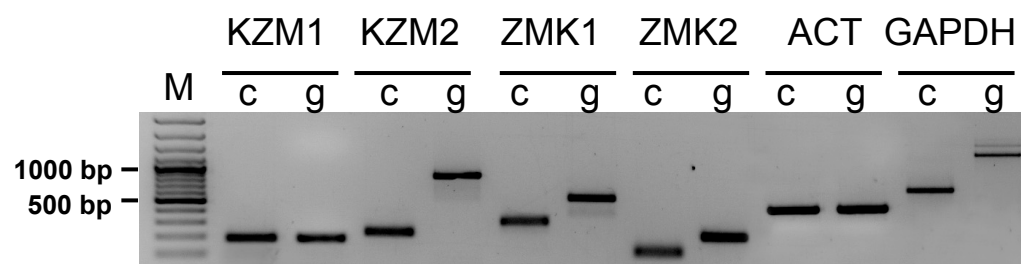

Supplement: Figure S6 — Expression analysis of potassium channels in pollen tubes of the maize inbred line A188. The expression of four known maize potassium channels was studied by RT-PCR using cDNA from pollen tubes as described in Supporting Information. KZM1 (AJ421640) & KZM2(AJ558238): K+ channels Zea mays 1 and 2; ZMK1 (Y07632) & ZMK2 (AJ132686): Zea mays K+ channel 1 and 2; RT-PCR controls: ACT: actin 81/83 (AAB40105); GAPDH (X07156): glycerinaldehyde 3-phosphate dehydrogenase. c: cDNA & g: genomic DNA was each used as a template, respectively. The size of various genomic PCR products (KZM2, ZMK1, ZMK2, and GAPDH) is larger than that of cDNAs indicating that the cDNA used as a template did not contain genomic DNA. M: 100 bp DNA ladder was used to visualize the length of the amplified DNA fragments. (0.21 MB PDF) [file pbio.1000388.s006.pdf]

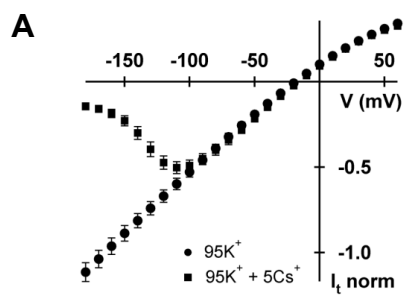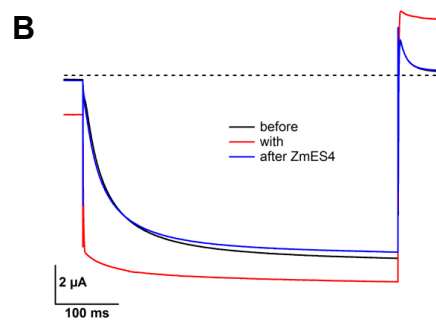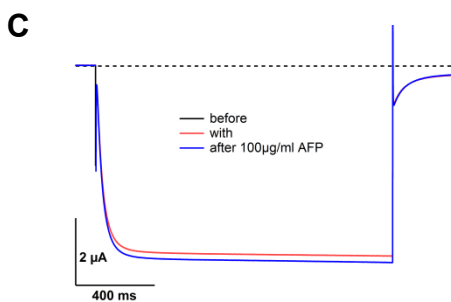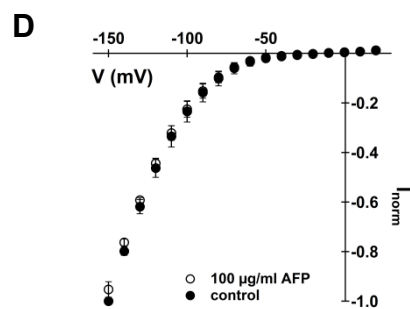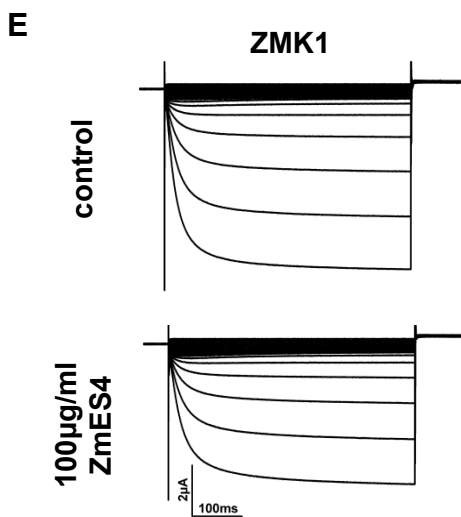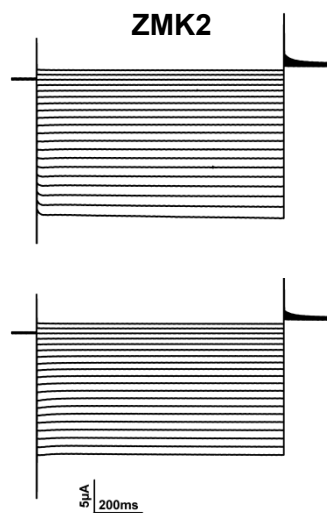

Supplement: Figure S7 — Reversibility of KZM1 activation by ZmES4 and inhibition of KZM1 currents by Cs+. (A) Instantaneous potassium currents (It) elicited by oocytes expressing KZM1 were inhibited by 5 mM Cs+ in a voltage dependent manner. Recordings were performed at membrane potentials in the range of +60 to −180 mV in a bath solution containing 95 mM KCl at pH 7.4. Data points of four independent experiments were normalized to the value at −160 mV in control solution. Error bars indicate the standard deviation. (B and C) Whole oocyte currents recorded in 100 mM KCl in the presence and absence of ZmES4 (B) or RsAFP2 (APP in C). Starting from a holding potential of −10 mV a single voltage pulse to −100 mV was applied. Due to the loss of voltage dependence, the application of ZmES4 altered the activation kinetics of KZM1 from a time-dependent activation to a instantaneous activation. Reversion to voltage dependence and time-dependent activation kinetics of KZM1 is observed after washout of ZmES4. In contrast RsAFP2 did not alter the activation kinetics of KZM1. (D) Current voltage relation of KZM1 steady state currents in response to 100 µg/ml RsAFP2. Recordings were performed as follows: currents were evoked upon voltage jumps in the range of +20 to −150 mV in a bath solution containing 100 mM KCl. Data points of four independent experiments were normalized to the value at −150 mV in control solution. Error bars indicate the standard deviation. (E) Application of ZmES4 did not affect kinetics of the maize potassium channels ZMK1 (left panel) or ZMK2 (right panel). Recordings were performed in 100 mM KCl, pH 7.4. Currents were monitored in response to voltage changes ranging from +20 to −170 mV in 10 mV decrements. (0.21 MB PDF) [file pbio.1000388.s007.pdf]
