# Supplementary material for: Defensin-Like ZmES4 Mediates Pollen Tube Burst in Maize via Opening of the Potassium Channel KZM1
Source: PLoS Biol. 2010 Jun 1;8(6):e1000388. doi: 10.1371/journal.pbio.1000388 (PMC2879413; doi:10.1371/journal.pbio.1000388)
Supplement: Figure S5 — Alignment of ZmES1 and ZmES4 precursors and close plant homologs. Close plant homologs; maize ZmLCR1: AY112374, ZmLCR2: AY108650, and ZmLCR3: AY105007, rice: OsLCR1: Os02g41910, OsLCR2: AL662939, and OsLCR3: Os03g03810; Arabidopsis thaliana: AtLCR69: At2g02100, AtLCR71: At2g02135, AtLCR72: At2g02140, and AtLCR74: At5g63660; spruce: PgLCR1: AAR84643 of Picea glauca and PaSPI1B: AAN40688 of Picea abies. Predicted N-terminal signal sequences are boxed. Eight conserved cysteine residues C1–C8 are highlighted in black, acidic amino acids in red, and basic amino acids in blue. Red lettering displays hydrophobic amino acids, aromatic amino acids are shown in pink, and serine as well as threonine in dark green. A consensus sequence of this knottin/defensin-subclass of cysteine-rich microproteins is given below mature protein sequences. Numbers at the right display length of precursor sequences. (0.40 MB PDF) [file pbio.1000388.s005.pdf]

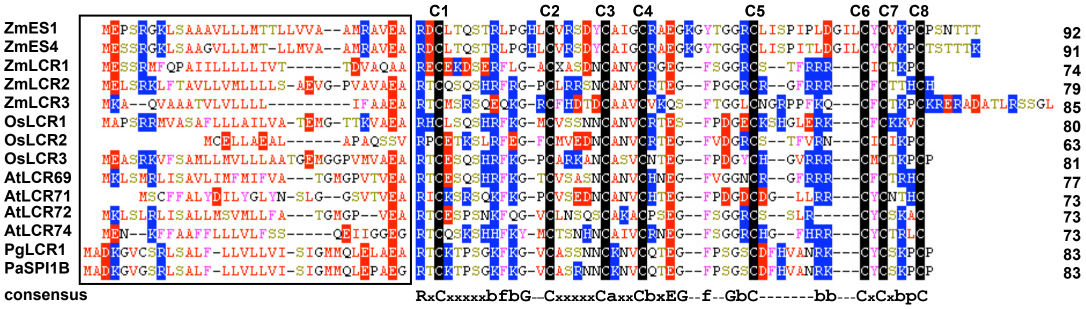
C1
C2
C3
C4
C5
C6
C7
C8
ZmES1 92
ZmES4 91
ZmLCR1 74
ZmLCR2 79
ZmLCR3 85
OsLCR1 80
OsLCR2 63
OsLCR3 81
AtLCR69 77
AtLCR71 73
AtLCR72 73
AtLCR74 73
PgLCR1 83
PaSPI1B 83
consensus
RxCxxxxxbfbG--CxxxxxCaxxCbxEG--f--GbC-------bb---CxCxbpC
