# Supplementary material for: Defensin-Like ZmES4 Mediates Pollen Tube Burst in Maize via Opening of the Potassium Channel KZM1
Source: PLoS Biol. 2010 Jun 1;8(6):e1000388. doi: 10.1371/journal.pbio.1000388 (PMC2879413; doi:10.1371/journal.pbio.1000388)
Supplement: Table S1 — Comparison of fertilization rates after in vitro pollination of WT and ZmES4-RNAi ovules using GUS-labeled pollen tubes. (0.06 MB DOC) [file pbio.1000388.s008.doc]

**Supporting Information**

**Supplement** **Table S1. Comparison of fertilization rates after *in vitro* pollination of WT and ZmES4-RNAi ovules using GUS-labelled pollen tubes.**

| **Experi-ment** | **h after pollination** | **Line** | **fertilized** | **unfertilized** | **Ovules ana-**  **lyzed (100%)** |
| --- | --- | --- | --- | --- | --- |
| #1  #2  #3  #4  #5 | 32 hap  29 hap  28 hap  27 hap  28 hap | ES4-RNAi #1(selfed) WT (A188) ES4-RNAi #1(selfed) WT (A188) ES4-RNAi #3(selfed) WT (A188) ES4-RNAi #1(selfed) WT (A188) ES4-RNAi #9(BC) WT (A188) | 13 (36.1%)  36 (73.5%)  19 (76%)  27 (90%)  26 (60.5%)  34 (85%)  26 (61.9%)  36 (94.7%)  25 (62.5%)  36 (94.7%) | 23 (63.9%)  13 (26.5%)  6 (24%)  3 (10%)  17 (39.5%)  6 (15%)  16 (38.1%)  2 (5.3%)  15 (37.5%)  2 (5.3%) | 36  49  25  30  43  40  42  38  40  38 |
| #6  #7  #8  #9 | 54 hap  53 hap  55 hap  55 hap | ES4-RNAi #1(selfed) WT (A188) ES4-RNAi#1(selfed) WT (A188) ES4-RNAi #1(selfed) WT (A188) ES4-RNAi #9(BC) WT (A188) | 10 (50%)  19 (79.2%)  15 (75%)  25 (92.6%)  13 (56.5%)  22 (88%)  11 (61.1%)  19 (95%) | 10 (50%)  5 (20.8%)  5 (25%)  2 (7.4%)  10 (43.5%)  3 (12%)  7 (38.9%)  1 (5%) | 20  24  20  27  23  25  18  20 |
